# Role of dislocation elastic field on impurity segregation in Fe-based alloys

**DOI:** 10.1038/s41598-020-80140-4

**Published:** 2021-01-19

**Authors:** I. Medouni, A. Portavoce, P. Maugis, P. Eyméoud, M. Yescas, K. Hoummada

**Affiliations:** 1 IM2NP, Faculté des Sciences de Saint-Jérôme case 142, Aix-Marseille University/CNRS, 13397 Marseille, France; 2 FRAMATOME, Développement (DTID) Et Ingénierie Mécanique (DTIM), 92084 Paris La Défense Cedex, France

**Keywords:** Materials science, Condensed-matter physics

## Abstract

Dislocation engineering in crystalline materials is essential when designing materials for a large range of applications. Segregation of additional elements at dislocations is frequently used to modify the influence of dislocations on material properties. Thus, the influence of the dislocation elastic field on impurity segregation is of major interest, as its understanding should lead to engineering solutions that improve the material properties. We report the experimental study of the elastic field influence on atomic segregation in the core and in the area surrounding edge dislocations in Fe-based alloys. Each element is found either to segregate in the edge dislocation core or to form atmospheres. The elastic field has a strong effect on the segregation atmosphere, but no effect on the dislocation core segregation. The theory is in good agreement with experiments, and should support dislocation engineering.

## Introduction

Dislocations play a major role in material properties, such as mechanical properties for structural materials^1,2^ and electrical or opto-electrical properties for functional materials^3–6^. Consequently, independently of the scale, from macroscopic to nanoscopic, dislocation engineering in crystalline materials is essential when engineering materials and their structures for any given application. One of the main tools allowing the influence of dislocation on material properties to be controlled, or at least modified, is impurity segregation. Consequently, investigation of impurity segregation at dislocations is a scientific and technological subject of high interest^7,8^. For example, boron (B) segregation at iron-aluminum dislocations, forming Cottrell atmospheres^9^ allowing tuning of Fe mechanical properties, has a major impact on the metallurgic industry. Likewise, hydrogen dislocation passivation in semiconductors plays a major role in the solar cell industry^10,11^. Though surface and grain boundary segregation can be observed and sometimes quantified using surface characterization tools, such as Auger electron spectroscopy^12^ and Nano secondary ion mass spectrometry^13,14^, quantification of dislocation atomic segregation is very challenging. Fortunately, the recent development (over the last few decades) of wide-field-of-view atom probe tomography (APT)^15,16^, as well as the development of APT data analysis techniques^17,18^, allow more chemical analyses to be performed at dislocations and in their surroundings^19–21^, with a spatial resolution allowing the dislocation core to be separated from its direct surroundings^22,23^. Indeed, dislocations are expected to influence impurity distribution for “chemical” reasons due to the different dangling bond energies between the matrix atoms and the impurities, and for “elastic” reasons due to the different sizes between the matrix atoms and the impurities, as well as due to the relaxation elastic field surrounding the dislocation. Dangling bond energy is expected to influence atomic segregation in the core of the dislocation, while elastic effects are expected to influence atomic segregation both in the dislocation core and in its surroundings. Dislocation segregation has been observed and studied essentially versus thermal treatments (fabrication process and material aging)^24,25^. However, the systematic study of the dislocation elastic field effect on impurity segregation has been rarely investigated experimentally. Nevertheless, this elastic field is expected to have a major impact on impurity segregation^26–28^.

### Theoretical considerations

**Figure 1:**
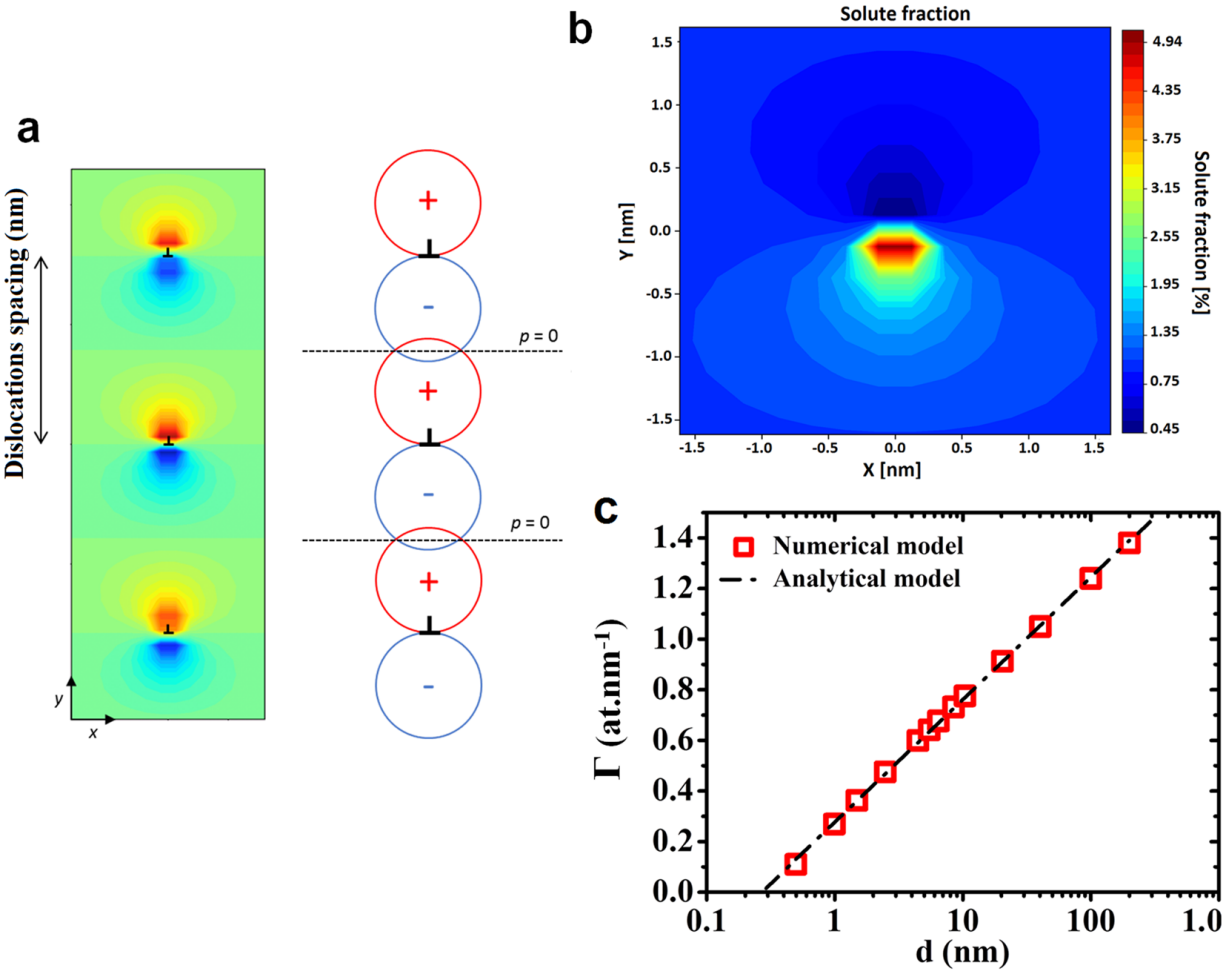
Theoretical dislocation elastic field influence on impurity segregation. (**a**) 1D periodical array of edge-dislocations of lattice parameter *d*. Left: pressure field numerically calculated using Eq. (1) (red for compression and blue for tension). Right: schematic showing the pressure annihilation at mid-distance planes due to the local interactions between tensile ( −) and compressive ( +) fields of two neighboring dislocations. (**b**) Equilibrium solute fraction field around one dislocation in the periodical array at *T* = 673 K, calculated numerically using Eqs. (1) and (2), with parameters corresponding to bcc-Fe with *C*_*0*_ = 1 at% and a dislocation spacing *d* = 3.2 nm. (**c**) Comparison between the solute Excess Number (${\Gamma }$) located on a dislocation calculated by numerical integration (Eqs. (1) and (2)) on a square mesh (dashed line) and calculated using the analytical model (Eq. (6)) as a function of log(d).

An ideal method allowing the dislocation elastic field effect on impurity segregation to be studied would consist in studying impurity segregation in a same matrix, containing several one-dimensional periodical arrays of same-type edge dislocations with different distances between dislocations (different periods), thermally annealed in the same conditions. For example, Fig. 1a presents a theoretical 1D array of periodically spaced edge dislocations located in a homogeneous matrix. In order to evaluate the stress field in the surroundings of the dislocation array, we used the Cottrell model^29^, which is applicable to isotropic solids. Although more sophisticated models can be used^30^, the Cottrell model presents the benefit of providing simple analytical formulas—after applying some algebra—allowing quantitative trends between experiment and simulation to be compared. We determined the expected stress distribution as a function of the position *x* (along the glide plane) and *y* (along the extra half-plane) from the dislocation core of a reference dislocation. The resulting pressure field *p* =  − 1/3 (*σ*_*xx*_ + *σ*_*yy*_ + *σ*_*zz*_) is a function of the reduced coordinates *x*/*d* and *y*/*d* such as:

1 $$p\left( {x,y} \right) = \frac{{Gb\left( {1 + \nu } \right)}}{{3\pi \left( {1 - \nu } \right)}}\frac{\pi }{d}\frac{{\sin \left( {2\pi y/d} \right)}}{{\cosh \left( {2\pi x/d} \right) - \cos \left( {2\pi y/d} \right)}}$$

with *d* the inter-dislocation spacing, *b* the Burgers vector length of the (identical) dislocations, and *G* and ν the shear modulus and Poisson’s ratio of the considered solid, respectively. The color gradients presented in Fig. 1a correspond to the pressure distribution (blue for tension and red for compression) numerically calculated using Eq. (1). The iso-pressure lines are not circles, contrary to the case of an isolated dislocation^29^. The pressure is null at the mid-distance planes where *y* =  ± *d*/2, and decreases approximately as exp(− 2π*x*/*d*) along the direction *x*, the long-range stress created by the dislocation array vanishing for *x* > *d*/2. Furthermore, the regions under compression stress and tensile stress between two neighboring dislocations interact and cause a decrease in the pressure field compared to an isolated dislocation^31^.

The goal of the present study is to experimentally determine the role of the elastic field on substitutional impurity segregation in the core and in the dislocation surroundings using APT measurements, and to compare the experimental observations to theory. Equation (1) can be used to determine impurity distribution in a 1D dislocation array based on the impurity relaxation volumes, using the McLean equation of segregation for a dilute solid solution^32,33^:

2 $$C\left( {x,y} \right) = C_{0} {\text{exp}}\left( {\frac{{ - p\left( {x,y} \right)\Delta V}}{{k_{B} T}}} \right)$$

The use of this equation is justified by the fact that solute fraction in the Cottrell atmosphere is low (less than a few percent): thus, solute–solute chemical interactions as well as site competition can been neglected, as a first approximation. Equation (2) allows the segregation fraction *C*(*x*,*y*) to be determined at a given temperature *T*, depending on the solute fraction *C*_0_ far from the dislocation and on the relaxation volume Δ*V* of the considered solute, knowing the local pressure variations (*k*_*B*_ is the Boltzmann constant). According to this equation, oversized impurity atoms (Δ*V* > 0) are expected to accumulate in the tensed regions (*p* < 0), while undersized atoms (Δ*V* < 0) are expected to accumulate in the compressed regions (*p* > 0). Figure 1b presents the two-dimensional (2D) solute equilibrium distribution calculated numerically using Eq. (1) and (2), and a 2D model. The model parameters were chosen to correspond to the bcc-Fe matrix (*G* = 82 GPa, *ν* = 0.28 eV, *b* = 0.2485 nm, Δ*V* = 0.001 nm^3^, and the atomic volume *V*_*0*_ = 0.0118 nm^3^ at^-1^) with *C*_*0*_ = 1 at% and *T* = 673 K. A square mesh of size $a = \left( {V_{0} } \right)^{1/3}$ and a dislocation spacing *d* = 3.2 nm were used. As expected, the oversized impurity atoms occupy the tensed matrix sites. APT is the only technique allowing for the quantitative measurement of the solute excess number per unit length of dislocation (${\Gamma }_{\text{exp}}$), which is the main parameter characterizing dislocation solute segregation at a given temperature^34^. Thus, in order to compare theoretical results to experimental measurements, the calculated data obtained from Eqs. (1) and (2) (Fig. 1b) should be used to determine the theoretical solute excess per unit length of dislocation ${\Gamma }$. ${\Gamma }$ can be calculated as follows:

3 $${\Gamma } = \frac{1}{{V_{0} }}\mathop \smallint \limits_{ - \infty }^{ + \infty } dx\mathop \smallint \limits_{ - d/2}^{ + d/2} dy\left( {C - C_{0} } \right)$$

The analytical integration of Eq. (3) is possible through the series development of Eq. (2) with respect to (*p*∆*V */*k*_*B*_*T*), and of Eq. (1) with respect to both *x*/*d* and *y*/*d*. However, the dislocation core region [+ *y*_*0*_/*d*, − *y*_*0*_/*d*] has to be excluded from the calculation in order to avoid the singularity located at *y* = 0 (Fig. 1b). The parameter *y*_*0*_ is on the order of one interatomic distance. Contrary to the case of an isolated dislocation, the integral converges due to the limited range of integration along *y* and to the fast decay of *C* along *x*. If only the first non-vanishing term of the expansion is kept, the solute excess is expressed as

4 $${\Gamma }_ = \frac{{\pi C_{0} }}{{2V_{0} }}\left( {\frac{{Gb\left( {1 + \nu } \right)}}{{3\pi \left( {1 - \nu } \right)}}\frac{\Delta V}{{k_{B} T}}} \right)^{2} {\text{ln}}\left( {\frac{d}{{y_{0} }}} \right).$$

Keeping only the first non-vanishing term of the expansion of Eq. (3) corresponds to neglecting the stress field of dislocations other than the nearest ones^31^. Equation (4) is similar to the Cottrell formula for an isolated dislocation, if *R*/*r*_0_ is substituted by *d*/*y*_0_ in the logarithm^35^. Upon higher-order expansion, the whole set of dislocations can be taken into account. The principal term of the series remains a logarithm, such that ${\Gamma }$ can be approximated to

5 $${\Gamma } \left( d \right) = A + B\, {\text{ln}}\left( {\frac{d}{{y_{0} }}} \right)$$

where *A* and *B* are fast-decaying functions of *d*. The comparison of Eq. (5) with the numerical integration on a square mesh shows that *A* and *B* can be taken to be constant with high accuracy (Fig. 1c). It results that the solute excess in the vicinity of the dislocations (excluding the dislocation core) can be written:

6 $${\Gamma } \left( d \right) = {\Gamma }^{0} {\text{ln}}\left( {\frac{d}{{d_{0} }}} \right)$$

where *d*_0_ and ${\Gamma }^{0}$ are constants. According to this equation, impurity segregation on isolated dislocations should be higher than on grouped dislocations due to the interaction of their elastic fields. Furthermore, in the case of a 1D regular array of dislocations characterized by a constant inter-dislocation distance *d*, impurity segregation should increase logarithmically with *d*.

### Experimental investigation of dislocation field effect on impurity segregation

As mentioned earlier, APT measurements allow impurity segregation to be quantitatively studied on dislocations^34^. Consequently, the experimental study of the dislocation elastic field influence on impurity segregation mainly depends on available samples containing reliable periodic dislocation arrays. Low-angle grain boundaries (GB), corresponding to a tilt angle *θ* less than 15°, are well known as being made of a regular 1D network of same-type edge-dislocations in bcc-Fe^36^. The dislocation spacing *d* in low-angle GBs is related to the GB tilt angle *θ* by the relation sin(*θ*/2) = *b*/2*d*^37^. Consequently, low-angle GBs could be used to experimentally determine the elastic field influence on impurity segregation in the core and in the dislocation surroundings. To this aim, we used a low-concentration-carbon steel annealed at 673 K for 30,000 h. This polycrystalline bcc-Fe sample was chosen because it contains several substitutional impurities such as molybdenum (Mo), manganese (Mn), nickel (Ni), phosphorus (P), chromium (Cr), and silicon (Si), as well as a high density of low-angle GBs of different tilts, corresponding to 1D edge-dislocation arrays exhibiting different dislocation spacings. Impurity segregation in the different low-angle GBs (i.e. in the different dislocation arrays) should allow reliable comparisons, since all the dislocation arrays are located in the same material and thus experienced the same thermal treatment. Furthermore, the impurity segregation is expected to be at thermodynamic equilibrium, due to the significant thermal budget used for the isothermal annealing.

**Figure 2:**
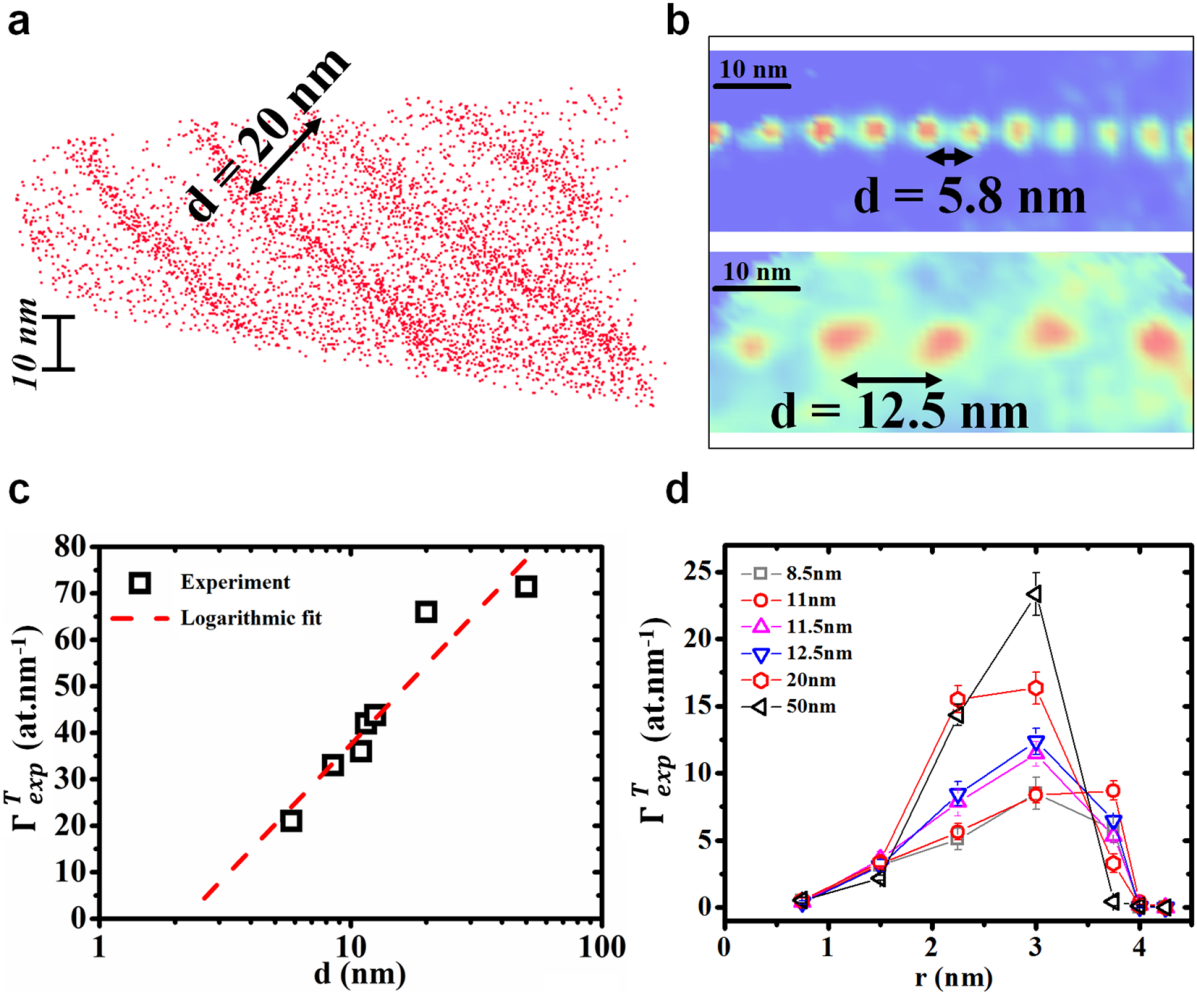
Experimental measurements of impurity segregation at edge-dislocations forming a regular 1D array characterized by an inter-dislocation distance *d*. (**a)** Volume of the bcc-Fe sample analyzed by APT, each dot corresponds to a single Mo atom. (**b**) Impurity 2D concentration maps determined on a plan perpendicular to the dislocation lines in two different dislocation arrays, with *d* = 5.8 and 12.5 nm. (**c**) Variations of the dislocation Total Excess Number (${\Gamma }^{\text{T}}_{\text{exp}}$) versus *d* compared to a logarithmic fit (dashed lines). (**d**) Variations of the dislocation ${\Gamma }^{\text{T}}_{\text{exp}}$ versus the radius *r* corresponding to the distance from the dislocation line in the cylindrical geometry, measured in different dislocation arrays exhibiting different *d* comprised between 8.5 and 50 nm.

Figure 2a presents a typical volume of the polycrystalline Fe sample analyzed by APT.

**Figure 3:**
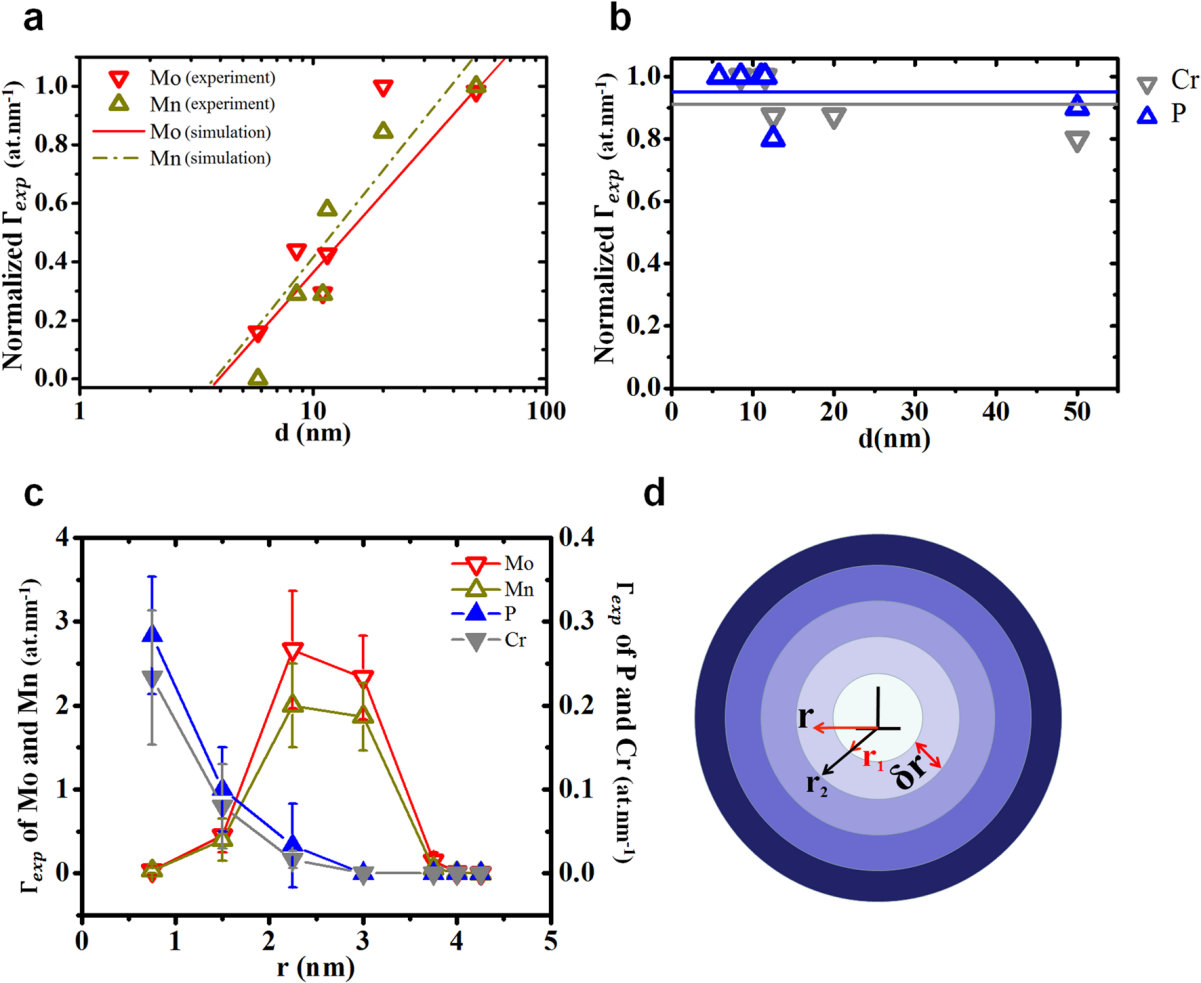
Experimental measurements of single element segregation at edge-dislocations forming a regular 1D array characterized by an inter-dislocation distance *d*. (**a**) Normalized Excess Number (${\Gamma }_{\text{exp}}$) variations at the dislocation versus *d* for Mo (down triangles) and Mn (up triangles) atoms compared to simulations (dotted line for Mo and solid line for Mn). (**b**) Normalized Excess Number (${\Gamma }_{\text{exp}}$) variations at the dislocation versus *d* for P (up triangles) and Cr (down triangles) atoms. (**c**) Variations of the dislocation ${\Gamma }_{\text{exp}}$ of Mo (open down triangles), Mn (open up triangles), P (solid up triangles), and Cr (solid down triangles) atoms versus the radius *r* corresponding to the distance from the dislocation line in the cylindrical geometry, measured in a dislocation array with *d* = 8.5 nm. (**d**) Schematic showing the top view of the concentric cylinder trenches used to determine the concentration-versus-radius profiles in APT volumes around each dislocation line (⊥).

Each dot corresponds to a single atom. Only Mo atoms are shown for clarity reasons. Four parallel lines can be observed, corresponding to impurity atomic accumulations along edge-dislocations (straight lines) forming a low-angle GB with a constant dislocation spacing *d* ~ 20 nm, which corresponds to a tilt angle of *θ* ~ 0.71°. In this case, the volume is oriented such that the decorated dislocations can be observed from a side-view through the entirevolume. Figure 2b shows two 2D concentration maps acquired from two different volumes, each containing a low-angle GB of a different tilt angle. The concentration maps integrate the concentrations of all the solute impurities on a 2D plane perpendicular to the dislocation lines, such that the decorated dislocations are observed in top view. The color gradient is proportional to the total impurity concentration (from blue for the lowest concentrations to red for the highest concentrations). The edge-dislocations form a regular array of parallel dislocation lines with an inter-dislocation spacing that can be considered as constant. The experimental excess number ${\Gamma }_{\text{exp}}$ was determined from APT data using the integral profile method^34,38^. However, due to the dislocation geometry (1D defect) compared to the interface geometry (2D defect), the integration was not performed along a classical 1D concentration-vs-depth profile measured perpendicularly to the defect^40^. The integration was performed along a 1D concentration profile in which concentrations are measured in concentric cylindrical trenches of constant thickness *δr* = 0.75 nm and of constant volume *δv* = 1 nm^3^ centered on the dislocation line (Fig. 3d). The choice of *δr* and *δv* was driven by the desire to resolve as much as possible the concentration variations around the dislocation line (small *δr*) compared to the resolution limitations due to (i) APT resolution^39–42^, (ii) the atomic density in the dislocation line vicinity, and (iii) the lengths of the dislocation lines. The result is given as a function of the average radius *r* of the considered cylindrical trench, corresponding to the average distance where the concentration was measured from the dislocation line, following the cylindrical symmetry (concentration-vs-radius profiles). The total excess number (${\Gamma }^{\text{T}}_{\text{exp}}$) was calculated as the sum of all the ${\Gamma }_{\text{exp}}$ of all the elements (Mo, Mn, Ni, P, Cr, Si and C) detected at each dislocation and averaged for each low-angle GB, in order to determine the variations of the ${\Gamma }^{\text{T}}_{\text{exp}}$ as a function of the average distance *d* measured between the dislocations in each GB. The values of the ${\Gamma }^{\text{T}}_{\text{exp}}$ are given in the last column of Tab. 1 versus the distance *d*. The Fig. 2c presents the experimental variation of the ${\Gamma }^{\text{T}}_{\text{exp}}$ versus *d*. As predicted by Eq. (6), these variations can be fit with a logarithmic law (dashed lines in Fig. 2c). The ${\Gamma }^{\text{T}}_{\text{exp}}$-vs-radius profiles measured for different inter-distances 8.5 ≤ *d* ≤ 50 nm (Fig. 2d) show different segregation behaviors between the dislocation core and its surroundings. The total impurity segregation level close to the dislocation core (smaller radius in Fig. 2d) is lower than that further away from the core, and exhibits a constant segregation level independent of *d*. The impurity segregation level variations versus *d* occur exclusively out of the dislocation core, in the dislocation surroundings up to *r* ~ 4 nm. In this region, impurity segregation forms atom atmospheres (or clouds) expected to be driven by the elastic field surrounding the dislocation line. Consequently, in this region the segregation level increases with *d* following a logarithmic law as predicted by Eq. (6), as the segregation level is constant close to the dislocation core. As presented in Fig. 2c,d, similar measurements concerning the collective segregation of the different solutes were performed for independent solute elements. The excess number (${\Gamma }_{\text{exp}}$) measured for each individual impurity versus *d* is given in Tab. 1. The different segregation behaviors observed between the core and the surroundings are actually linked to the nature of the segregating element. For example, the segregation of Mo and Mn atoms on the dislocations varies with the distance between dislocations as proposed by Eq. (6) (Fig. 3a), while the segregation of P and Cr atoms is constant and independent of *d* (Fig. 3b). These observations, compared to the results presented in Fig. 2c,d, lead to the conclusion that P and Cr atoms occupy the dislocation core, while Mo and Mn atoms form Cottrell’s atmospheres surrounding the dislocation line. In order to confirm this hypothesis, the ${\Gamma }_{\text{exp}}$-vs-radius profiles of these four elements were determined using the same technique as for the ${\Gamma }^{\text{T}}_{\text{exp}}$-vs-radius profiles presented in Fig. 2d, based on concentration-vs-radius profiles determined from concentric cylindrical trenches of same thickness and same volume centered on the dislocation lines (Fig. 3d). The ${\Gamma }_{\text{exp}}$-vs-radius profiles of Mo, Mn, P, and Cr measured on dislocations separated by a distance *d* = 8.5 nm are presented in Fig. 3c. The spatial distribution difference between the elements is clearly observed. Indeed, P and Cr atoms mainly occupy the core of the dislocations with ${\Gamma }_{\text{exp}}$ ~ 0.25 at nm^−1^, while Mo and Mn atoms are mainly located at 2.5 nm from the dislocation core, with an excess number about one order of magnitude higher ${\Gamma }_{\text{exp}}$ ~ 2.5 at nm^−1^. This difference of excess numbers between the core and the surrounding of the dislocations is in agreement with the number of atomic sites available for segregation in these two regions. For example, considering the Fe bcc structure, the number of sites in the dislocation core can be estimated to be 4.3 sites nm^−1^ for a dislocation line in the direction [1 1 -2]. P and Cr are thus expected to have saturated the dislocation core, since the sum of their ${\Gamma }_{\text{exp}}$ gives ~ 5 at nm^−1^. However, as shown by Cottrell and Bilby^29^, the long-range stress is negligible for a distance larger than *d*. This is why the number of sites occupied by Mo and Mn atoms is significantly smaller than the number of available sites. The resulting solute radial distribution exhibits a peak at a finite distance from the dislocation core, in agreement with the numerical integration of our 2D model (not shown). For example, the Mo and Mn ${\Gamma }_{\text{exp}}$ = 3.6 at nm^−1^ and the number of available substitutional sites is ~ 2850 sites nm^−1^ for *d* = 5.8 nm. The experimental variations of Mo and Mn excess numbers with the inter-dislocation distance *d* can be compared to the theoretical expectations comparing the data presented in Fig. 3a and the theoretical ${\Gamma }$ calculated with Eq. (4). In this equation, the unique unknown parameter is the solute relaxation volume Δ*V*. The best fit (Fig. 3a) was obtained with the values Δ*V*^Mo^ = 6 Å^3^ and Δ*V*^Mn^ = 3 Å^3^.

In order to confirm the accuracy of these relaxation volumes, Density Functional Theory-based calculations were performed using the VASP software with spin-polarization^43–47^ aiming to determine the relaxation volumes of Mo, Mn, P, and Cr in bcc-Fe. Adopting a calculation procedure similar to that presented in Ref. ^48^, a set of Special Quasirandom Structures^49^ (SQS) with the ATAT code^50^ was first built for each solute *X* (*X* = Mo, Mn, P, and Cr), with the respective stoichiometry Fe_128-*n*_*X*_*n*_ (*n* = 1, 2, 3, and 4). After the relaxation of the structures under zero-stress with the conjugate-gradient algorithm of VASP, a linear fit was performed on the average supercell volume per concentration, as a function of the number *n* of atoms *X* in the supercells. Finally, the leading coefficients corresponding to the relaxation volumes Δ*V*^Mo^ =  + 5.5 Å^3^, Δ*V*^Mn^ =  + 2.5 Å^3^, Δ*V*^P^ =  − 1.0 Å^3^, and Δ*V*^Cr^ =  + 2.0 Å^3^ were extracted. These SQS-DFT results confirm that the relaxation volume of Mo is about twice that of Mn, as shown by the mean-field approach. Furthermore, the results are in good agreement with theoretical and experimental works reported in the literature (see Tab. 2): Δ*V* is negative for smaller p-block elements such as P, and positive for transition elements such as Mo, Mn, and Cr. One can note that i) the elements found in the dislocation core can exhibit both negative and positive relaxation volumes, and ii) the elements found in the neighboring dislocation line exhibit significantly larger relaxation volumes. In order to match the experimental conditions, ${\Gamma }$ was calculated at *T* = 673 K, with *C*_0_^Mo^ = 0.16 at% and *C*_0_^Mn^ = 1.1 at%. The calculation results (solid line for Mo and dotted line for Mn) are superimposed on the experimental data (down triangles for Mo and up triangles for Mn) in Fig. 3a. Experimental and calculated results are in reasonable agreement.

### Outlook

Available theory based on elasticity cannot predict impurity segregation behavior in a dislocation core, but can predict a strong influence of the dislocation elastic field on impurity segregation. Experiments obtained on Fe-based alloys show that the elements segregated in the dislocation core or forming the atmosphere around the dislocation line are different. The dislocation elastic field is shown to exert a strong influence on the formation of segregation atmospheres, with in our case a segregation level multiplied by five when the inter-dislocation spacing is multiplied by six (8.5 ≤ *d* ≤ 50 nm). Furthermore, in the case of a 1D dislocation array of lattice parameter *d*, the segregation level varies according to a logarithmic law of *d* as theoretically proposed in the present work. However, the dislocation long-range elastic field is found to have no effect on atomic segregation in the dislocation core, and impurities segregated in the dislocation core can have opposite elastic properties (i.e. positive or negative relaxation volume). These experimental results, coupled with theoretical considerations, bring new insights into the driving forces controlling impurity segregation either in the dislocation core or the dislocation surroundings, and are expected to lead to dislocation engineering strategies based on thermodynamic or thermomechanical processes coupled with impurity doping strategies allowing for the improvement of Fe-based alloys properties in a large range of applications.

## Methods

### Material

The material analyzed in this study is a bainitic low alloy 18MND5 steel. The steel was subjected to a long-term aging heat treatment at 400 °C for 30,000 h. The nominal composition given by the founder of the low alloy steel (LAS) is given in Supplementary Table 1 in the additional information file.

**Table 1:** Dislocation excess number (${\Gamma }_{\text{exp}}$) of different solute elements present in the low-carbon-concentration bcc-Fe sample (i.e. C, Mn, Mo, Ni, P, Cr, and Si) and their total excess number (${\Gamma }^{\text{T}}_{\text{exp}}$) measured in different 1D edge-dislocation arrays exhibiting different inter-dislocation distance *d*. C is the only interstitial specie, all the other elements are substitutional in the Fe bcc lattice.

*d* (nm)	C (nm^−1^)	Mn (nm^−1^)	Mo (nm^−1^)	Ni (nm^−1^)	P (nm^−1^)	Cr (nm^−1^)	Si (nm^−1^)	${\Gamma }^{\text{T}}_{\text{exp}}$ (nm^−1^)
5.8	7.8 ± 0.5	/	3.6 ± 0.4	6.3 ± 0.7	3 ± 0.3	/	/	21 ± 1.8
8.5	12 ± 0.4	7.2 ± 0.3	9.9 ± 0.1	/	2.4 ± 0.3	2.4 ± 0.2	/	33 ± 1.3
11	5.4 ± 0.6	7.5 ± 0.2	6.6 ± 0.3	10.5 ± 0.2	3 ± 0.1	2.4 ± 0.2	/	36 ± 1.6
11.5	7.8 ± 0.6	14.4 ± 0.3	9 ± 0.2	6.3 ± 0.4	3 ± 0.2	2.1 ± 0.2	/	42 ± 1.9
12.5	5.4 ± 0.5	22.5 ± 0.4	9.6 ± 0.3	/	2.4 ± 0.4	/	3 ± 0.5	43.5 ± 2.1
20	20.4 ± 2	21 ± 0.2	22.5 ± 09	/	/	2.1 ± 0.5	/	66 ± 3.6
50	3.9 ± 0.5	24.9 ± 0.3	22.2 ± 0.2	7.8 ± 0.4	2.7 ± 0.2	2.1 ± 0.2	7.5 ± 0.2	71.4 ± 2

### Atom probe tomography analyses

Atom probe tomography (APT) sample preparation was carried out using a dual beam SEM/FIB FEI Helios NanoLab 600. A standard lift-out followed by annular milling was used to prepare the tips. APT measurements were performed using a CAMECA LEAP 3000X HR setup in electric mode. The analyses were performed at 80 K, under a pulse rate of 200 kHz. The pulse fraction was set to 20% and the evaporation rate to 0.2% per pulse.

### Ab initio calculations

Four Special Quasirandom Structures were constructed using the *mcsqs* modulus^51^ of ATAT code^50^, for each of the following stoichiometries: ${\text{Fe}}_{{\text{128 - n}}} {\text{X}}_{{\text{n}}}$ (${\text{n}} = 2,3,4$). The supercell volumes were obtained by zero-stress relaxation using the conjugate-gradient algorithm of VASP software^43–47^, with spin-polarization. We used PAW-PBE functionals and 400 eV plane-wave energy cut-off, with $6 \times 6 \times 6$ Monkhorst–Pack grids including Γ-point. Due to the preferential antiparallel spin coupling of these dilute solutes in bcc iron^52,53^, magnetic moments of solutes where initialized antiferromagnetically ($- 2\mu_{B}$ for the solute and $+ 2.5\mu_{B}$ for iron). We then plotted the average supercell volume per concentration with respect to the number of atoms *X* in supercells. Linear fits were performed on the five stoichiometries ${\text{Fe}}_{{\text{128 - n}}} {\text{X}}_{{\text{n}}}$ (${\text{n}} = 0,1,2,3,4$), except for Mn, for which only the three low-concentrated values (*n* = 0, 1, 2) were used, in order to avoid the magnetocrystalline effect at high concentrations^52^.

For comparison with literature data (see Table 2), the relaxation volumes were extracted from calculated (Exact Muffin-Tin Orbitals Coherent Potential Approximation)^54^ and experimental (X-ray diffraction)^48,55–58^ evolutions of lattice parameters $a$ with fraction of solute $x$, using the equality $\Delta V = V_{0} \cdot \frac{3}{{a_{0} }} \cdot \frac{da}{{dx}}$ , wherein $a_{0}$ (resp. $V_{0} = \frac{{a_{0}^{3} }}{2}$) denotes the lattice parameter (resp. atomic volume) of pure iron.

**Table 2:** Computed relaxation volumes Δ*V*(Å^3^): SQS-DFT, mean-field approach and literature data.

	SQS-DFT	Mean-field	Literature	
			Calculated	Experimental
P	− 1.0	n.a	n.a	− 1.56^46^, − 1.1^49^
Cr	+ 2.0	n.a	+ 2.3^47^	+ 0.52^46^, + 1.6^50^
Mn	+ 2.5	± 3.0	+ 2.6^32^, + 2.5^48^, + 2.4^47^	+ 0.58^46^, + 1.1^51^
Mo	+ 5.5	± 6.0	n.a	+ 3.25^46^, + 3.8^50^

## Supplementary information

Supplementary Information 1.

## Data Availability

All the necessary data is available in the main text or the supplementary materials. Any other relevant data are also available upon request from A.P., with the exception of the full original POS files obtained from APT measurements, since these sets of data contain additional information unrelated to the subject of this study, which are the property of FRAMATOME.
