# Supplementary material for: Role of dislocation elastic field on impurity segregation in Fe-based alloys
Source: Sci Rep. 2021 Jan 19;11:1780. doi: 10.1038/s41598-020-80140-4 (PMC7815746; doi:10.1038/s41598-020-80140-4)
Supplement: Supplementary file 1 — Supplementary Information 1. [file 41598_2020_80140_MOESM1_ESM.pdf]

Role of dislocation elastic field on impurity segregation in Fe-based alloys

I. Medouni[1,2], A. Portavoce[1]*, P. Maugis[1], P. Eyméoud[1], M. Yescas[2], K. Hoummada[1]

[1]*IM2NP, Aix-Marseille University/CNRS, Faculté des Sciences de Saint-Jérôme case 142, 13397 Marseille, France.*
[2]*FRAMATOME, Développement (DTID) et Ingénierie Mécanique (DTIM), 92084 Paris La Défense CEDEX.*

[*]Corresponding author: alain.portavoce@im2np.fr

Supplementary Text

The bulk nominal composition of the low alloy steel (LAS) is given in **Supplementary Table 1**, and **Supplementary Table 2** presents the concentration of the different elements detected by APT in the bulk of the sample.

In order to increase the probability of seizing a low angle grain boundary (LAGB) in the APT volumes, the sample regions analyzed by APT were selected combining electron backscatter diffraction (EBSD) measurements and dual beam focus ion beam (FIB) in situ preparation. **Supplementary Fig. 1** shows a typical EBSD image acquired on the LAS sample. The yellow dash circles highlight the regions of interest from were APT samples were prepared. They correspond to regions containing LAGBs separating grains of similar crystallographic orientations.

Supplementary Fig. 2 presents the APT data obtained on one of the seven edge dislocation arrays (#1 to #7) used in this study to investigate dislocation elastic field effect on impurity segregation. 2D concentration maps of the segregating elements, determined over planes perpendicular to the dislocation lines, were used to correctly position a cylindrical volume (perpendicular to the plane of the 2D maps) around each dislocation line (cylinder centered on the dislocation line) in which the concentration of the elements located in the vicinity of the considered dislocation line was quantified. The diameter of this cylindrical volume was kept constant and equal to 6 nm for all the measurements. The concentrations of all the segregating elements on the dislocation lines were found to be constant up to this diameter. Furthermore, this value allows the overlapping between the Cottrell distributions of neighboring dislocation lines to be prevented in all the measurements we performed. Iso-concentration surfaces were also used to detect the dislocation lines and to correctly perform the concentration measurements on the dislocation lines.

Supplementary Fig. 3 Illustrates the technique used to determine the segregation excess number (Γ_{exp}) on a single dislocation line. As already mentioned, the concentration or the number of segregated atoms on each dislocation line present in a given array was determined using a cylindrical volume of diameter 6 nm centered on the dislocation line. The Γ_{exp} on each dislocation line was thus calculated as the difference between the atom number measured in the dislocation line vicinity and the atom number measured in the matrix divided by the cylinder length (at nm^{-1}). The atom number measured in the matrix, far from the dislocation array, was determined using the same cylindrical volume. The Γ_{exp} values obtained for each element on each dislocation line in a same array was averaged over all the dislocation lines, in order to determine the average Γ_{exp} corresponding to the considered array, characterized by the average distance between dislocations in the array.

Supplementary Fig. 4 illustrates the technique used to determine the concentration-vs-radius profiles, which were used to determine the Γ_{exp}-vs-radius profiles. Due to the geometry of the defect (line), the concentration of the considered element was measured in concentric cylindrical trenches of constant thickness (Δr = 0.75 nm) and of constant volume (Δv = 1 nm^3) centered on the dislocation line, as shown in the top view presented in **Supplementary Fig. 4b**. Thus, the concentration (**Supplementary Fig. 4c**) can be plotted versus the distance r from the dislocation line (**Supplementary Fig. 4d**). In the example presented in **Supplementary Fig. 4**, the P and Mo atoms are clearly forming a core/shell structure, the P atoms occupying the core of the dislocation line, and the Mo atoms surrounding the P distribution at a distance from the dislocation core of ~ 2.5 nm.

	**Fe**	**C**	Mn	Si	**Mo**	**Cr**	Co	Ni	**Cu**	**Al**	**P**	**S**
**at.%**	96.39	0.872	1.48	0.413	0.285	0.163	0.010	0.68	0.086	0.037	0.013	0.01
**wt.%**	96.63	0.188	1.46	0.208	0.491	0.152	0.011	0.712	0.098	0.018	0.0067	0.0066

Supplementary Table 1: Nominal composition of the LAS 18MND5.

	**Fe**	**C**	Mn	Si	**Mo**	**Cr**	Co	Ni	**Cu**	**Al**	**P**	**S**
**at.%**	96,88	0,02	1.16	0.46	0,15	0.11	0.010	0.9	0.03	0.02	0.03	0.01

Supplementary Table 2: Nominal composition of the LAS 18MND5 measured by APT.

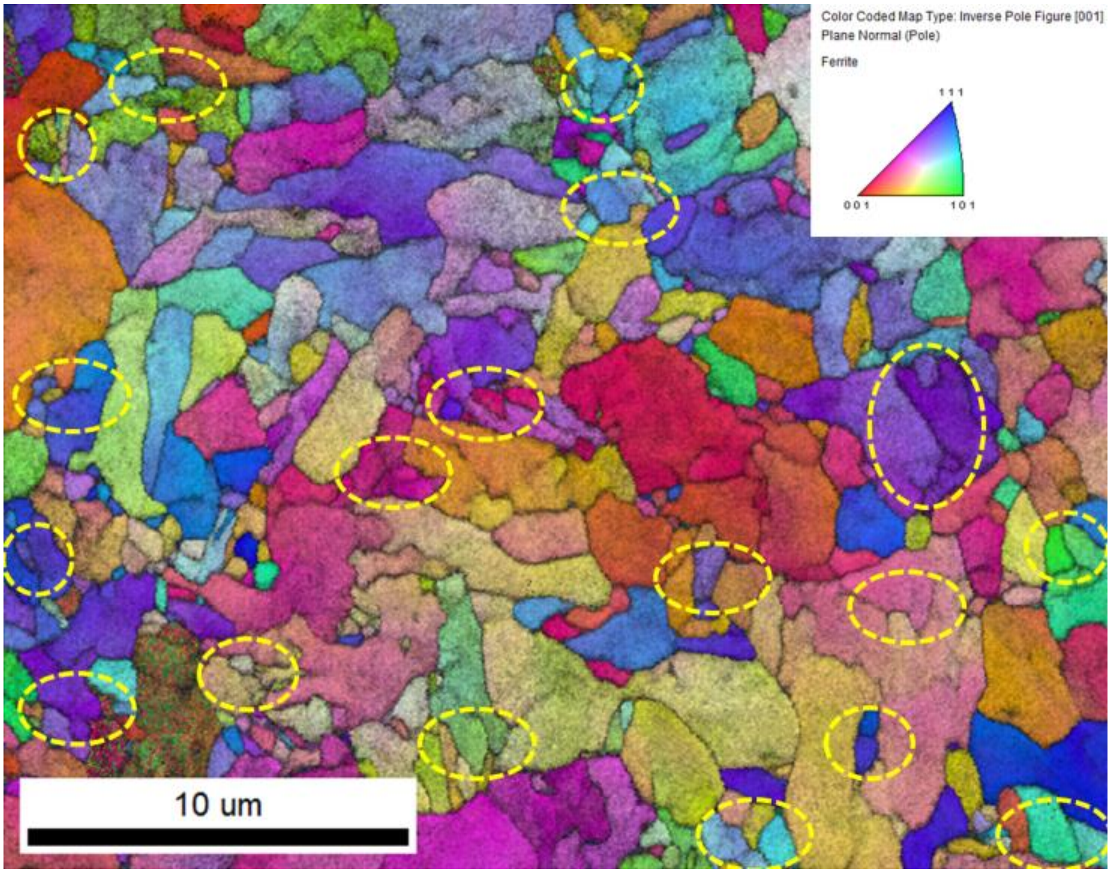

Supplementary Fig. 1. EBSD inverse pole figure and image quality grain orientation mapping. Yellow dash circles highlight regions with LAGB separating grains with similar crystallographic orientations.

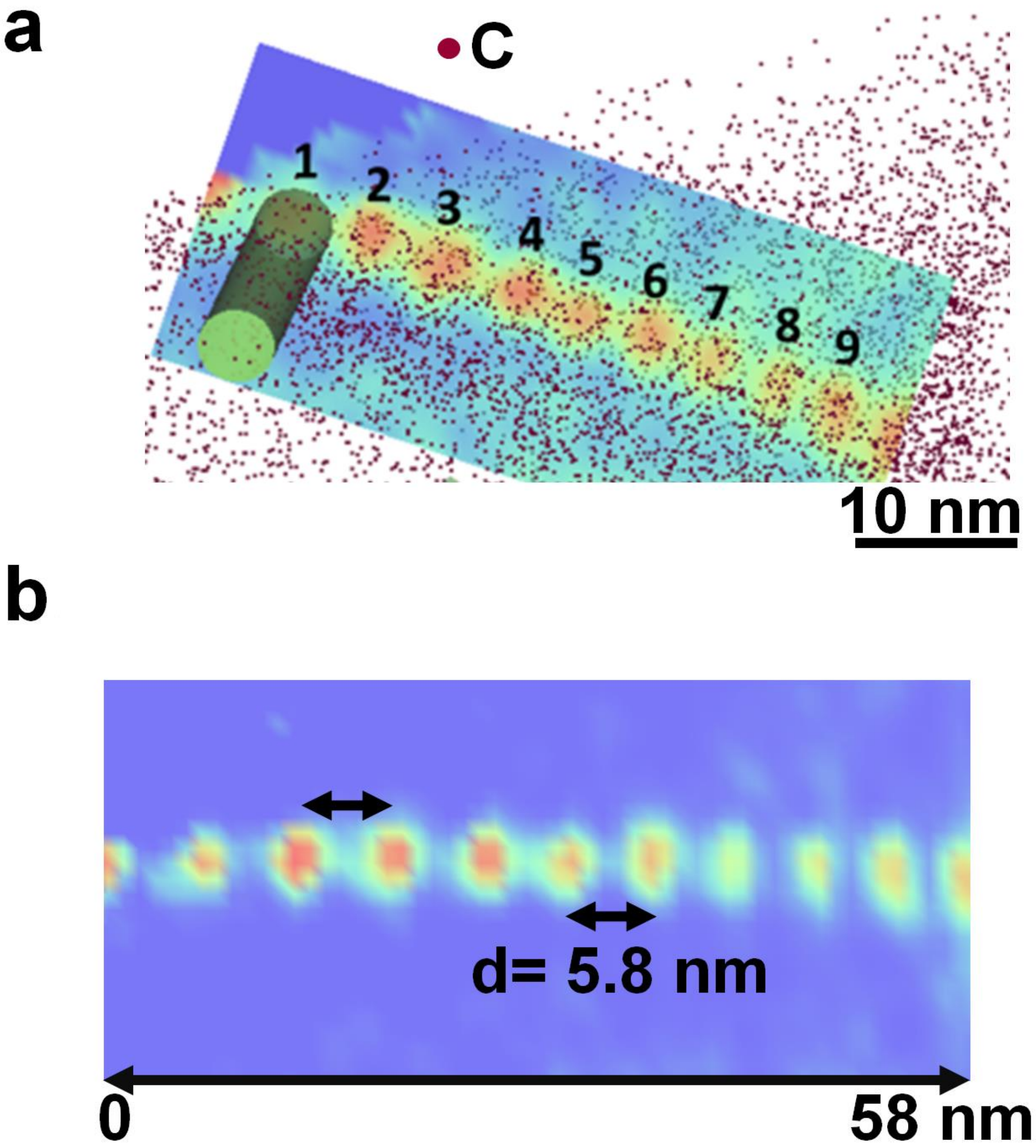

Supplementary Fig. 2. Dislocation array #1 corresponding to an average dislocation interspacing (i.e. lattice parameter) *d* = 5.8 nm: a, APT volume (each dot corresponds to a single atom) with the superimposition of a 2D concentration map of all the segregating elements determined on a plane perpendicular to the dislocation lines (blue for low concentrations and red for high concentrations), the cylinder corresponds to the volume used to quantify the concentration of the elements on each dislocation line, it is perpendicular to the plan of the 2D map. **b**, enlargement of the 2D concentration map shown in **a**, presented in plan-view, 11 dislocation lines are observed.

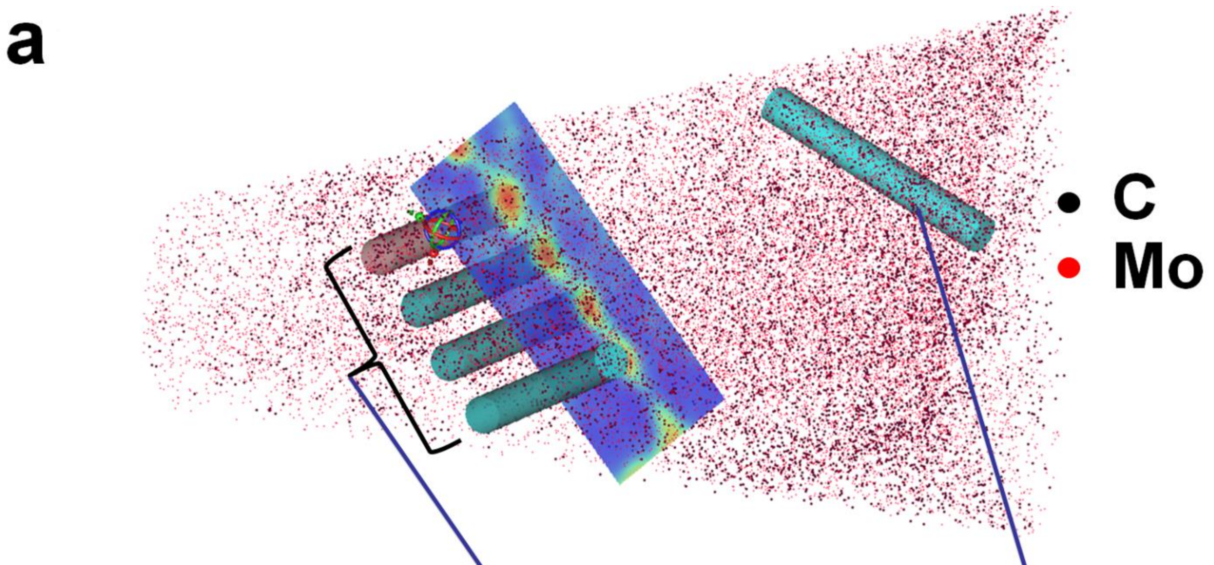

b

Atom	Atom count at dislocation lines	Atom count in ferrite matrix
Mn	408.75	264
Ni	406.8	302
Si	206	180
Mo	138	40
C	125.5	46
P	65	14
Cr	47	29.75

Supplementary Fig. 3. Illustration of the technique used to determine the excess number (Γ_{exp}) on a single dislocation line. The values obtained for each dislocation line in a same array was averaged over all the dislocation lines in order to determine the average Γ_{exp} of the given array: **a**, APT volume (each point corresponds to a single atom) corresponding to the dislocation array #3, the same cylindrical volume was centered on each considered dislocation line (cylinders perpendicular to the 2D map in **a**) and each element present in the cylinder was counted considering the mass spectrum obtained in the cylinder. The same cylinder was used to determine the quantities of the same elements present in the matrix, far from the GB (isolated cylinder in the APT volume). **b**, table showing the average number of atoms of each segregated elements measured in the dislocation line vicinity and in the matrix far from the dislocation lines.

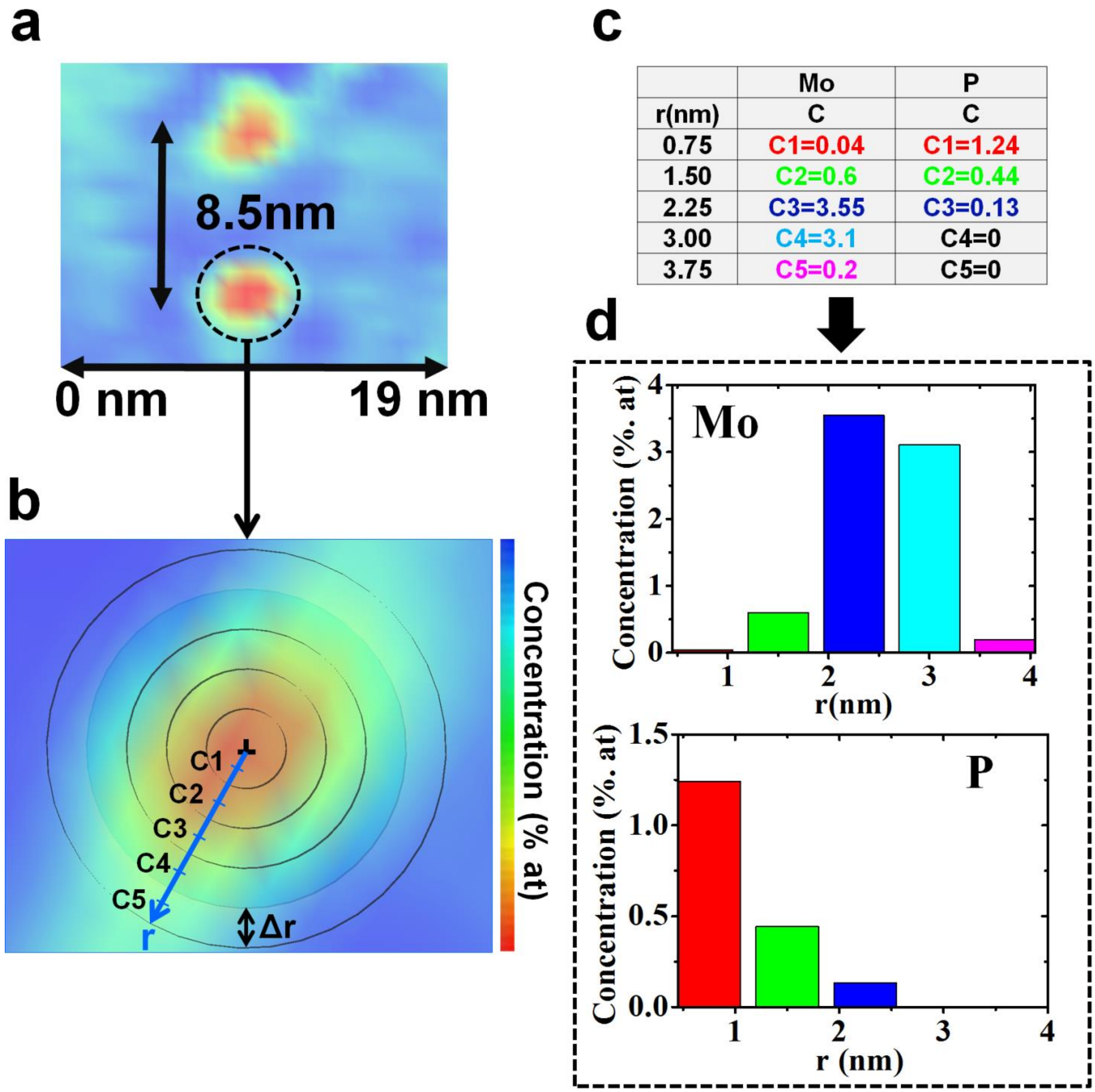

Supplementary Fig. 4. Illustration of the technique used to determine the concentration-vs-radius profiles that were used to determine the Γ_{exp}-vs-radius profiles: **a**, plan-view 2D concentration map of all the segregating elements determined on a plane perpendicular to the dislocation lines in the array #2 (blue for low concentrations and red for high concentrations). **b**, enlargement of the 2D distribution measured on the dislocation line circled in **a** with the top view of the different concentric cylindrical volumes of same thickness Δr = 0.75 nm in which the Mo and P concentrations were measured. **c**, table giving the Mo and P concentrations measured in each concentric cylindrical volumes shown in top view in **b**. **d**, Mo and P concentration variations versus the distance r from the dislocation line (concentration-vs-radius profiles) deduced from the data presented in **c**.
